# A Text Messaging Intervention for Coping With Social Distancing During COVID-19 (StayWell at Home): Protocol for a Randomized Controlled Trial

**DOI:** 10.2196/23592

**Published:** 2021-01-14

**Authors:** Caroline Astrid Figueroa, Rosa Hernandez-Ramos, Claire Elizabeth Boone, Laura Gómez-Pathak, Vivian Yip, Tiffany Luo, Valentín Sierra, Jing Xu, Bibhas Chakraborty, Sabrina Darrow, Adrian Aguilera

**Affiliations:** 1 School of Social Welfare University of California Berkeley Berkeley, CA United States; 2 School of Public Health University of California Berkeley Berkeley, CA United States; 3 Centre for Quantitative Medicine Duke-National University of Singapore Medical School Singapore Singapore; 4 Data Science Program Division of Science and Technology Beijing Normal University-Hong Kong Baptist University United International College Zhuhai, Guangdong China; 5 Department of Statistics and Applied Probability National University of Singapore Singapore Singapore; 6 Department of Biostatistics and Bioinformatics Duke University Durham, NC United States; 7 Department of Psychiatry and Behavioral Sciences University of California San Francisco San Francisco, CA United States; 8 Zuckerberg San Francisco General Hospital San Francisco, CA United States

**Keywords:** COVID-19, mental health, depression, reinforcement learning, microrandomized trial

## Abstract

**Background:**

Social distancing is a crucial intervention to slow down person-to-person transmission of COVID-19. However, social distancing has negative consequences, including increases in depression and anxiety. Digital interventions, such as text messaging, can provide accessible support on a population-wide scale. We developed text messages in English and Spanish to help individuals manage their depressive mood and anxiety during the COVID-19 pandemic.

**Objective:**

In a two-arm randomized controlled trial, we aim to examine the effect of our 60-day text messaging intervention. Additionally, we aim to assess whether the use of machine learning to adapt the messaging frequency and content improves the effectiveness of the intervention. Finally, we will examine the differences in daily mood ratings between the message categories and time windows.

**Methods:**

The messages were designed within two different categories: behavioral activation and coping skills. Participants will be randomized into (1) a random messaging arm, where message category and timing will be chosen with equal probabilities, and (2) a reinforcement learning arm, with a learned decision mechanism for choosing the messages. Participants in both arms will receive one message per day within three different time windows and will be asked to provide their mood rating 3 hours later. We will compare self-reported daily mood ratings; self-reported depression, using the 8-item Patient Health Questionnaire; and self-reported anxiety, using the 7-item Generalized Anxiety Disorder scale at baseline and at intervention completion.

**Results:**

The Committee for the Protection of Human Subjects at the University of California Berkeley approved this study in April 2020 (No. 2020-04-13162). Data collection began in April 2020 and will run to April 2021. As of August 24, 2020, we have enrolled 229 participants. We plan to submit manuscripts describing the main results of the trial and results from the microrandomized trial for publication in peer-reviewed journals and for presentations at national and international scientific meetings.

**Conclusions:**

Results will contribute to our knowledge of effective psychological tools to alleviate the negative effects of social distancing and the benefit of using machine learning to personalize digital mental health interventions.

**Trial Registration:**

ClinicalTrials.gov NCT04473599; https://clinicaltrials.gov/ct2/show/NCT04473599

**International Registered Report Identifier (IRRID):**

DERR1-10.2196/23592

## Introduction

### Background

The current COVID-19 pandemic not only poses a large threat to physical health but also has detrimental consequences for mental health. Social distancing is a crucial intervention to slow down person-to-person transmission of this infectious disease. However, culminating research shows that it also has unintended consequences for large groups of the population: increased anxiety, depression, and stress [1,2]; decreased physical activity [3,4]; and lower sleep quality [5]. In the United States, vulnerable populations from low-income backgrounds, people of color, and Spanish speakers are more likely to work in jobs where they are at higher risk of contracting COVID-19 [6]. In part because of this, these groups experience disproportionately worse mental health outcomes [6,7].

The current situation calls for new and innovative digital methods to reach vulnerable populations [8]. Text-messaging interventions, which can be implemented during social distancing, have previously demonstrated effectiveness in behavioral health promotion and disease management [9]. They are also suitable for low–digital literacy populations and underserved groups [10]. For instance, our own Health Insurance Portability and Accountability Act (HIPAA)-approved texting platform, HealthySMS, has shown high acceptability and engagement among low-income English and Spanish speakers in California [11-13].

We developed text messages based on cognitive behavioral therapy to help people cope with the stress and anxiety of COVID-19 social distancing. Messages are developed within two different categories: behavioral activation (BA) [14] and other skills, more typical of psychoeducation for improving mood [15]. BA messages include prompts to increase BA and decrease avoidance of anxiety-inducing situations. Other skills focused on changing thinking patterns and tips about sleep, self-care, and breathing exercises; see examples of messages in Table 1. We will distribute this text messaging system to a wide group of individuals in the United States via social media advertisements. Further, we designed these messages both in English and in Spanish, enabling the program to reach a diverse group of people. Mobile health interventions are less often designed for Spanish speakers.

### Objective

The main purpose of this study, which is called the StayWell at Home study, is to examine whether automated text messages will improve depression and anxiety symptoms and enhance positive mood. Additionally, we will compare the effectiveness of sending messages on a random schedule using a microrandomized trial (MRT) design [16], further referred to as *uniform random* (UR), or sending messages via a *reinforcement learning* (RL) algorithm on the overall change in depression and anxiety symptoms and daily mood during the 60-day study. Finally, within the microrandomized group, we will examine which types of text messages are more effective in helping people increase their positive mood. We will examine the hypotheses discussed in the following two sections.

### Primary Analysis

We hypothesize that participants will show improvements in depression symptoms, measured using the 8-item Patient Health Questionnaire (PHQ-8); anxiety symptoms, measured using the 7-item Generalized Anxiety Disorder (GAD-7) scale; and daily mood during the 60-day study. We will conduct a pre-post comparison among all participants.

We hypothesize that the participants in the group receiving RL will have a greater decrease in depressive symptoms and anxiety and a greater daily increase in mood ratings during the 60-day study than participants in the UR group (ie, randomized design).

### Secondary Analysis

We hypothesize that we will find differential effects on mood ratings for the two categories of messages and different timings (ie, microrandomized design).

## Methods

### Design

This study has various designs: (1) a pre-post comparison, in which we assess changes in depression and anxiety for all patients before and after the intervention; (2) a randomized controlled trial with two groups, RL and UR; and (3) an MRT, only within the UR group.

**Figure 1:**
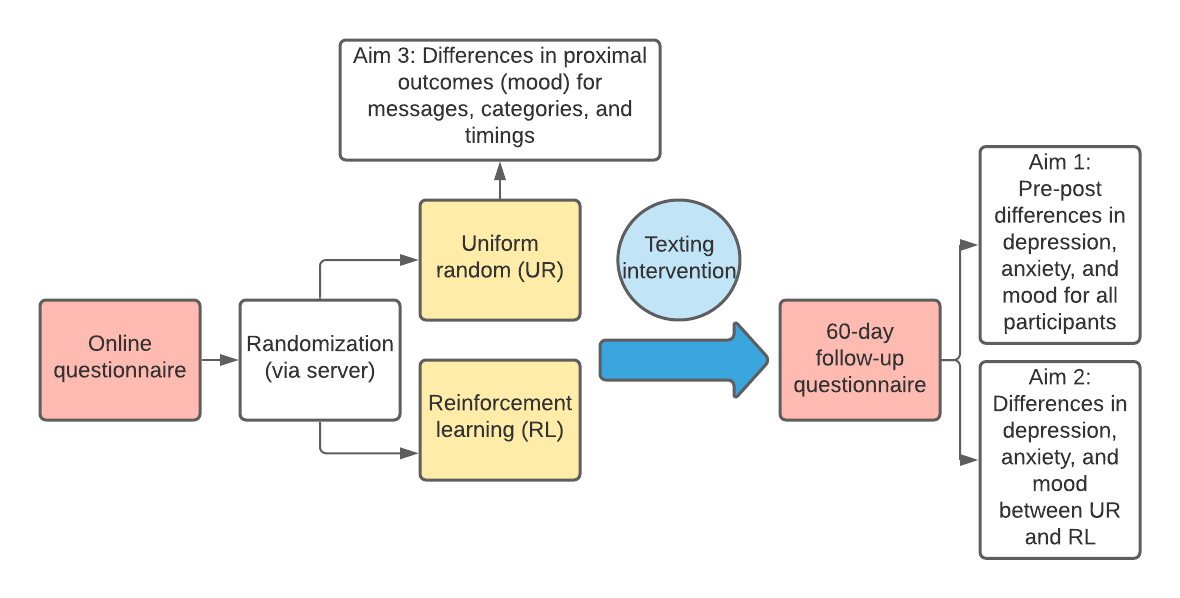
StayWell at Home study design.

Randomization will be performed as block randomization with a 1:1 allocation. Participants will be automatically randomized into groups through our secure server during onboarding of the study, ensuring allocation concealment. Participants will be informed of the nature and frequency of the messages they will be receiving. They will be blinded to their group randomization. Further, if messages are not sent out appropriately, research assistants will contact the developer to address errors (eg, when individuals do not receive messages, or they receive messages out of the specific time bounds). Throughout the study, the researchers will check whether the randomization of messages is functioning adequately approximately once every two months. The necessity of these steps makes it infeasible to blind the researchers. Microrandomization will happen automatically on a daily basis through our secure server. We used the SPIRIT (Standard Protocol Items: Recommendations for Interventional Trials) checklist when writing this protocol [17]. Figure 1 shows our study design.

### Recruitment

This is a fully remote trial. We will recruit on social media platforms, such as Facebook, Twitter, and Craigslist, and through university websites (ie, University of California [UC] Berkeley and UC San Francisco). Our posts and ads will be designed by the research team to target low-income, vulnerable populations across the United States. We will utilize the *detailed targeting* feature on Facebook to select the group of people to whom we want to show our ads. We will recruit in both English and Spanish.

The Facebook posts and ads will be informed by user-centered design (UCD) methods, including implementation of user personas in recruitment efforts. User personas, a common UCD tool [18], consist of fictional characters that represent our target populations. The user personas will include English speakers and Spanish speakers of various demographic groups. Each ad will contain a title and picture, followed by a reason for participating in the study and enrollment details. We will rely on Facebook’s built-in algorithms to present the most relevant ad version to each viewer.

### Inclusion Criteria

We will include adults 18 years or over who have a functioning mobile phone and who speak English and/or Spanish. We will exclude participants who use an online text messaging app, as this is more prone to online scams and fraud (eg, individuals creating fake accounts to receive reimbursements). Through targeted ads, we will make concerted efforts to recruit vulnerable populations, such as low-income individuals and people of color, who are disproportionately impacted by COVID-19 in the United States.

### Measures

For our primary outcomes, we will administer a survey at baseline and at 60-day follow-up, which includes the PHQ-8 [19] and GAD-7 [20]. In addition to these questionnaires, we will also ask open-ended questions to assess how participants are impacted by COVID-19. Questionnaire data will be stored on UC Berkeley’s Qualtrics platform. Our secondary outcome, daily mood ratings, will be collected via text message on a daily basis and stored on the HealthySMS platform. The project coordinator and research assistants will be responsible for managing patient data collection. Once data from all participants are collected, they will be stored on UC Berkeley’s Secure Box, a secure cloud-hosted platform. See Table 1 [21,22] for all included questionnaires and timing of their administration.

**Table 1:** Questionnaires included in the StayWell at Home study and timing of administration.

Questionnaire^a^	Baseline	Follow-up
PHQ-8 (8-item Patient Health Questionnaire)^b^	X^c^	X
GAD-7 (7-item Generalized Anxiety Disorder) scale^b^	X	X
COVID-19 questions	X	X
System Usability Scale^d^	N/A^e^	X

^a^The measures were taken from validated questionnaires in English and Spanish.

^b^The PHQ-8 depression scale and the GAD-7 anxiety scale were not modified.

^c^X indicates that the measure was administered at this time point.

^d^The System Usability Scale [21] was modified to decrease literacy levels, using the Flesch-Kincaid readability test [22].

^e^N/A: not applicable; the measure was not administered at this time point.

### Procedure

#### Baseline Assessment

Interested subjects will be sent to the designated Qualtrics platform to verify that their mobile phone number and ZIP Code are based in the United States. We will also determine human identity using a built-in CAPTCHA.

The project coordinator and/or research assistants will email each subject a one-time use personalized link. Subjects will click on the designated link taking them to a Qualtrics questionnaire. Here, they will give their informed consent and indicate whether they are over 18 years old. Thereafter, we will collect all baseline survey measures of interest as well as patient demographics. Upon survey completion, participants will be automatically enrolled onto the text messaging platform.

#### Intervention

##### Text Messages

We will send participants supportive text messages for a period of 60 days. These text messages include tips about BA and other coping skills to deal with worries and stress. The text messages used in this effort were based on core principles of evidence-based interventions for depression and anxiety and focus on rapid adoption of new behavior change strategies. Messages are balanced so that half the messages are related to BA and half are framed around other skills (see Table 2). Participants will receive one of these messages within three different time windows per day, between 9 AM and 6 PM. Participants will be sent a message asking them to rate their mood on a scale of 1 to 9, with 9 being the best mood, 3 hours after receiving the BA or skills message. These text messages were based on previous work conducted by SD and AA [23,24] and were edited by the study team members. Examples of BA and skill-based text messages are shown in Table 2.

**Table 2:** Examples of StayWell at Home text messages.

Message category	Example text messages
Behavioral activation	“Make a list of people that make you happy. Commit to reaching out to at least one of them each day this week.” “If there is something you have always wanted to do, like learning to play the guitar or painting, try a YouTube video today for learning a new skill.”
Other skills and coping	“Ugh. Sheltering in place is hard. Take some time to feel angry or sad or whatever you are feeling.” “If you are feeling a bit more sad or stressed right now, you are not alone. This is a hard time, but you can do this!”

##### Messaging Platform

We will use a text messaging platform, HealthySMS, developed by AA, to send text messages and manage participant responses back to our system. HealthySMS has been successfully used with various low-income, adult populations in English and Spanish [11-13].

##### Uniform Random Policy

This study design is an MRT [16], where every day during the study treatment, allocation is characterized by a full factorial design with a total of two factors representing supportive *messages* and the *time frame* when the message was sent. The *messages* factor has two levels, and the *time frame* factor has three levels (ie, 9 AM-12 PM, 12 PM-3 PM, and 3 PM-6 PM). Each participant will be rerandomized to a new combination of *messages* and *time frame* every day. The study is set up so that each day participants will be randomized to receive one message out of the message categories (ie, BA vs other skills), constituting a multilevel MRT design with probabilities of 0.5 for the message categories and 0.33 for the timing. Thus, every participant will receive one BA or skill message per day and one mood check-in message.

**Figure 2:**
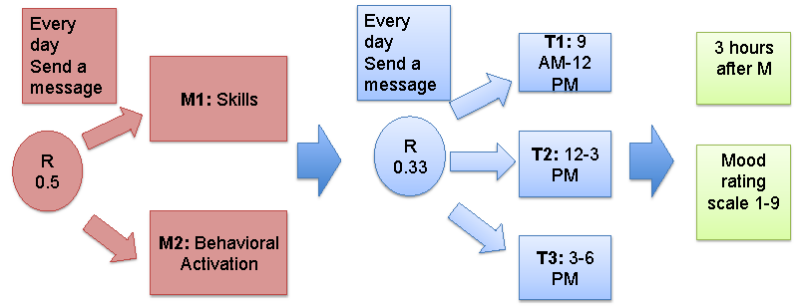
Microrandomized trial design of the StayWell at Home study. M: message; T: time point.

This design allows us to understand the type and timings of text messages that most improve participants’ mood, which is a secondary aim of this study. MRTs enable the testing of specific intervention components, while still allowing for the evaluation of a causal, average treatment effect of the intervention [16]. Figure 2 shows our MRT design.

##### Reinforcement Learning Policy

We employ a learned decision mechanism for the timing and type of text message. The RL algorithm learns from previous data to maximize an increase in participants’ mood using a linear regression model that is updated every morning, comparable to previous work in mobile health [25]. The learning data include which message category was previously sent and at what time, days since messages were sent, participants’ mood after the messages, and the day of the week (ie, Monday-Sunday). We employ two different learned decision mechanisms—one for the type of message and one for the timing of the message—using two separate linear regression models.

Both groups (ie, UR and RL) will receive the same messages. However, for the UR group, the type and timing of text messages will be randomly selected, whereas for the RL group, they will be selected by a learning algorithm. We will compare the effect of sending text messages by a random schedule with sending text messages chosen by the RL algorithm. This allows us to assess whether using RL to adapt the messaging scheme is more effective than a random messaging schedule. Additionally, we will be able to evaluate the effect of the individual intervention components over time, within an MRT design.

Participants in both groups can reply “STOP” or “PARAR” if they wish to stop receiving messages at any time during the study within 60 days.

### Statistical Analysis Plan

#### Primary Analysis

Paired *t* tests will be used to detect the improvement in depression score (ie, PHQ-8) and anxiety score (ie, GAD-7) from baseline to follow-up measured 60 days later. Two-sample *t* tests will be used to examine the difference in improvements between the UR and RL groups. The mood ratings of each participant will be collected each day over the 60-day study period. We will also compare the average of daily mood rating improvements compared to baseline (ie, each consecutive daily measure minus the baseline measure) between these two groups using a longitudinal data analysis approach (eg, generalized estimating equation [GEE]). We will not compare the baseline characteristics between the samples in each arm. This practice could potentially be misleading because any differences would be due to chance [26-28].

#### Secondary Analysis

For the UR intervention group, the differences in proximal outcome (ie, daily mood rating assessed 3 hours after the message sent) between both message categories (ie, BA versus coping skills) or among the time windows (ie, reference level versus the other three levels) will be examined using the weighted and centered least-squares (WCLS) method for longitudinal data analysis under the multilevel MRT design proposed by Xu et al [24]. This method is similar to the GEE approach. The independent working correlation matrix will be adopted. The covariates include days in study (ie, from 1 to 60 days), day of the week (ie, Monday-Sunday), intervention component (ie, message or time window), and the interaction term between days and intervention component. The trend of the intervention effect over days can be constant, linear, or quadratic. The message or the time window component categories will be converted to dummy variables, and each of these will be centered by the corresponding randomization probabilities (ie, 0.5 and 0.33 for each level of the message and time components, respectively).

The mood rating changes from baseline will be categorized into binary outcomes (ie, high [greater or equal to the median of the whole sample] or low [less than the median]). The WCLS estimator under the MRT design for binary outcome proposed by Qian et al [29] can be applied to the message component (ie, two-level intervention). For the timing (ie, three-level) component, we will propose the novel WCLS method by combining the ideas of both Xu et al [24] and Qian et al [29]. This method can also be extended to model the mood ratings as ordinal outcome variables. 

#### Sensitivity Analysis

The following sensitivity analyses will be or can be performed:

1. Both the primary and secondary analyses will be repeated based on the participants with at least 45 out of 60 (75%) days of data.
2. The change of the secondary outcome will be imputed using the last-observation-carried-forward method if either the corresponding pretest or posttest value is missing.
3. We will conduct the secondary analyses—the effects of message categories and timings—in the RL group.
4. An interaction between the components of the message and time window can be considered in the GEE model for the secondary hypothesis. This interaction term allows us to examine what type of message, sent at what time of the day, leads to the highest increase in daily mood ratings.

#### Normality Assumption Check

The normality assumptions for both primary and secondary outcomes will be checked by quantile-quantile plots. If normality fails, then the outcome variable will be taken as logarithm transformation (ie, log). We will add 0.5 to the zero-change value for either the depression or anxiety score, or the mood rating before applying the log transformation. 

### Power Analysis

We will perform sample size calculation at the usual 80% power at 5% level of significance.

#### Primary Analysis

The Cohen methods [30] were used to calculate sample sizes for primary hypotheses. At a medium standardized effect size (ie, Cohen *d*=0.5), a total sample size of 64 is required to detect an improvement of either the depression or anxiety score from baseline to 60-day follow-up, and a sample size of 128 to detect differences between the UR and RL groups.

#### Secondary Analyses

Using the GEE-based sample size calculation method [31,32] with a small standardized effect size (ie, Cohen *d*=0.2), with the correlation coefficients of 0.2 and 0.4 among the daily mood rating improvements compared to baseline, sample sizes of 84 and 161 are required to detect a group effect for either the UR or RL group, respectively, randomly allocated at baseline. Assuming 15% of the participants will drop out before the end of the study, a sample size of 190 is required for each group.

At a small standardized effect size (ie, Cohen *d*=0.1), with a constant trend of intervention effect over days, sample sizes of 55 and 76 are required to detect the average daily causal effects of message and time window, respectively, for the UR intervention group, using the WCLS-based sample size calculation method proposed by Xu et al under the multilevel MRT design [33]. Assuming each participant has an expected 70% response rate to the sent messages and 15% of the participants are expected drop off before the end of the study, a sample size of 126 is recommended for the UR group. 

Since our primary aim is to detect differences between the UR and RL groups for depression and anxiety scores, we aimed to include at least 128 participants. However, since our goal is also to provide a service during this unprecedented COVID-19 pandemic, we will continue to make the program available and recruit participants until at least April 2021 or for as long as funding allows.

### Engagement Measures

In addition to the measures mentioned above, we will also explore measures of engagement, such as response rates to the mood messages and the BA and skills messages as well as usability data, assessed by the System Usability Scale. This will help us to improve future iterations of the texting program.

### Compensation

Participants will receive no compensation for participation in the baseline part of the study. They will receive US $20 for completion of the 60-day follow-up questionnaire.

### Data Statement

We will submit study results for publication in peer-reviewed journals and for presentations at national and international meetings. We will aim to publish all findings in open access journals when possible or in other journals with a concurrent uploading of the manuscript content into PubMed Central for public access. Curated technical appendices, statistical code, and anonymized data will become freely available from the corresponding author upon request.

### Potential Harms

Participants will be instructed to contact the researchers if their phones are lost or stolen to ensure that we stop sending messages to them. Our study website will serve as the way to control which participants receive messages and when. The server receiving data from participants (ie, text responses) is hosted behind a UC San Francisco firewall in a secure location subject to health care–grade security measures, including strict firewalls, intrusion detection, and active monitoring by study and university staff.

### Ethics and Dissemination

The informed consent form for this study can be found in Multimedia Appendix 1. All protocol amendments will be communicated for approval to the UC Berkeley Committee for the Protection of Human Subjects (CPHS). We will ensure that our text messaging content is publicly available through a Creative Commons licensing agreement. The HealthySMS system is available for use upon request.

## Results

The UC Berkeley CPHS approved this protocol in April 2020 (No. 2020-04-13162) and the trial was registered at ClinicalTrials.gov (NCT04473599). Our enrollment started on April 17, 2020, and will continue to April 2021. As of August 24, 2020, we have enrolled 229 participants, of whom 218 were English speaking and 11 were Spanish speaking.

## Discussion

### Overview

The COVID-19 pandemic and the measures taken to combat it, such as social distancing, can take a large toll on mental health, exacerbating stress and symptoms of anxiety and depression. This study aims to assess the effect of a text messaging tool for improving mental health by providing daily text messages based on BA and skill building. We expect the text messages sent to all participants in this study to improve participant well-being, as measured by depression, anxiety symptoms, and daily mood ratings, by encouraging healthy behaviors and improving coping skills.

The COVID-19 pandemic has demonstrated the need for affordable, scalable, and effective digital mental health tools [8]. Here, we provide such a tool to a wide group of individuals and examine its effectiveness.

In addition to the primary study outcome of mental health, we will also be able to assess the feasibility and challenges of deploying a large-scale public health text messaging intervention completely remotely. The COVID-19 pandemic and measures to combat the spread of the virus led to the necessity to conduct many operations online, including research. Online surveys, online consent forms, virtual online recruitment strategies, and mobile or internet interventions and programs are crucial during this time.

This study will provide important insights and practical tools on remote recruitment with English and Spanish speakers in the United States. This will also allow us to write and disseminate guidelines for other researchers in this space. Knowledge on the careful implementation of user-centered programs is now more important than ever.

Of note, our recruitment up to this point has been significantly slower for Spanish-speaking participants. Previous work also reported that recruiting Hispanics or Latinxs who speak little or no English into randomized trials is challenging [34], and online recruitment may be even more difficult because of digital literacy issues. We hope to increase the recruitment rate of our monolingual Spanish-speaking population by continuously improving the personalization of our ads on websites, such as Facebook, which has been identified as an effective strategy for online recruitment with Spanish speakers [35].

One advantage of text messaging–based interventions is the ability to easily incorporate machine learning algorithms into the research design and test whether this approach improves effectiveness. RL algorithms have the potential to greatly contribute to the effectiveness of digital mental health studies as well as to the personalization and tailoring of these studies [36,37].

Though there is a tremendous interest in the use of machine learning techniques to improve mobile health interventions, not many studies have examined the feasibility and effectiveness of these approaches. Our unique design allows us to assess the added benefit of using RL on participant outcomes, as opposed to a random messaging schedule. While the microrandomized UR group facilitates the estimation of causal effects, the participants of that group do not benefit from that knowledge. In contrast, the participants of the RL arm get allocated to empirically better-performing messages with higher probabilities as the trial progresses and new knowledge accrues. Thus, the RL arm is an outcome-adaptive MRT design, which learns online, with the randomization probability of the intervention messages being adjusted according to the participant’s responses. This design is more participant-centric than the standard MRT, which learns offline, with equal and fixed randomization probabilities over the study period. Thus, a comparison of UR versus RL arms is a comparison between these two design approaches.

Additionally, MRTs are a novel and currently underutilized study design. Typical MRTs consider binary-level components (ie, control versus intervention); however, in this study, we instead use a unique multilevel MRT design, where there are more than two levels for the intervention [33]. This study will, thus, also provide various methodological contributions, especially to the digital health literature. Results from the MRT design will allow us to optimize our text messaging intervention and serve as preliminary evidence for a just-in-time adaptive intervention (JITAI). A JITAI is a type of personalized intervention that aims to provide the right type and amount of support at the right time and is adapted to an individual’s state [38]. This will be relevant information for optimizing this text messaging stress prevention app. 

### Limitations

There are disadvantages to fully online recruitment. For instance, participants may perceive a lack of connection to the research without contact with the researcher and, therefore, show lower engagement [39]. Furthermore, online recruitment comes with risks of fraudulent activity. In addition, our monetary incentive may lead to a selection of a sample mostly motivated by financial incentives. We aimed to minimize this potential bias by only providing the reimbursement at the end of the study. We also aimed to design a messaging bank with content relevant for a broad demographic group. Thus, the content might not be adequately tailored toward specific subgroups (eg, people with chronic physical diseases or severe mental illness). Finally, we use a multilevel MRT design as opposed to contrasting sending a message with not sending a message, which is a more common design. However, by using this design, we will not be able to assess the pooled effectiveness of sending any message versus no message.

### Conclusions

This study will examine whether automated supportive text messages will improve depression, anxiety, and mood of a broad community sample in a fully remote trial. In addition, we will assess whether using an RL algorithm to personalize messages is more effective than randomly selected messages. Overall, results will contribute to our knowledge of effective psychological tools to alleviate the negative effects of social distancing.

## Appendix

Multimedia Appendix 1 Informed consent form.

## Abbreviations

BA: behavioral activation
CPHS: Committee for the Protection of Human Subjects
GAD-7: 7-item Generalized Anxiety Disorder
GEE: generalized estimating equation
HIPAA: Health Insurance Portability and Accountability Act
JITAI: just-in-time adaptive intervention
MRT: microrandomized trial
PHQ-8: 8-item Patient Health Questionnaire
RL: reinforcement learning
SPIRIT: Standard Protocol Items: Recommendations for Interventional Trials
UC: University of California
UCD: user-centered design
UR: uniform random
WCLS: weighted and centered least squares

## References

[1] Rajkumar RP (2020). COVID-19 and mental health: A review of the existing literature. Asian J Psychiatr.

[2] Czeisler MÉ, Lane RI, Petrosky E, Wiley JF, Christensen A, Njai R, Weaver MD, Robbins R, Facer-Childs ER, Barger LK, Czeisler CA, Howard ME, Rajaratnam SM (2020). Mental health, substance use, and suicidal ideation during the COVID-19 pandemic - United States, June 24-30, 2020. MMWR Morb Mortal Wkly Rep.

[3] Engle S, Stromme J, Zhou A (2020). Staying at home: Mobility effects of COVID-19. SSRN.

[4] Tison G, Avram R, Kuhar P, Abreau S, Marcus G, Pletcher M, Olgin JE (2020). Worldwide effect of COVID-19 on physical activity: A descriptive study. Ann Intern Med.

[5] Cellini N, Canale N, Mioni G, Costa S (2020). Changes in sleep pattern, sense of time and digital media use during COVID-19 lockdown in Italy. J Sleep Res.

[6] Webb Hooper M, Nápoles AM, Pérez-Stable EJ (2020). COVID-19 and racial/ethnic disparities. JAMA.

[7] Bibbins-Domingo K (2020). This time must be different: Disparities during the COVID-19 pandemic. Ann Intern Med.

[8] Figueroa CA, Aguilera A (2020). The need for a mental health technology revolution in the COVID-19 pandemic. Front Psychiatry.

[9] Willcox JC, Dobson R, Whittaker R (2019). Old-fashioned technology in the era of "bling": Is there a future for text messaging in health care?. J Med Internet Res.

[10] Schueller SM, Hunter JF, Figueroa C, Aguilera A (2019). Use of digital mental health for marginalized and underserved populations. Curr Treat Options Psychiatry.

[11] Aguilera A, Bruehlman-Senecal E, Demasi O, Avila P (2017). Automated text messaging as an adjunct to cognitive behavioral therapy for depression: A clinical trial. J Med Internet Res.

[12] Aguilera A, Berridge C (2014). Qualitative feedback from a text messaging intervention for depression: Benefits, drawbacks, and cultural differences. JMIR Mhealth Uhealth.

[13] Figueroa CA, DeMasi O, Hernandez-Ramos R, Aguilera A (2021). Who benefits most from adding technology to depression treatment and how? An analysis of engagement with a texting adjunct for psychotherapy. Telemed J E Health.

[14] Farchione TJ, Boswell JF, Wilner JG (2017). Behavioral activation strategies for major depression in transdiagnostic cognitive-behavioral therapy: An evidence-based case study. Psychotherapy (Chic).

[15] Donker T, Griffiths KM, Cuijpers P, Christensen H (2009). Psychoeducation for depression, anxiety and psychological distress: A meta-analysis. BMC Med.

[16] Klasnja P, Hekler EB, Shiffman S, Boruvka A, Almirall D, Tewari A, Murphy SA (2015). Microrandomized trials: An experimental design for developing just-in-time adaptive interventions. Health Psychol.

[17] Chan A, Tetzlaff J, Altman D, Laupacis A, Gøtzsche PC, Krleža-Jerić K, Hróbjartsson A, Mann H, Dickersin K, Berlin JA, Doré CJ, Parulekar WR, Summerskill WSM, Groves T, Schulz KF, Sox HC, Rockhold FW, Rennie D, Moher D (2013). SPIRIT 2013 statement: Defining standard protocol items for clinical trials. Ann Intern Med.

[18] Norman DA, Draper SW (1986). User Centered System Design: New Perspectives on Human-Computer Interaction.

[19] Kroenke K, Spitzer RL, Williams JBW (2001). The PHQ-9: Validity of a brief depression severity measure. J Gen Intern Med.

[20] Spitzer RL, Kroenke K, Williams JBW, Löwe B (2006). A brief measure for assessing generalized anxiety disorder: The GAD-7. Arch Intern Med.

[21] Bangor A, Kortum PT, Miller JT (2008). An empirical evaluation of the System Usability Scale. Int J Hum Comput Interact.

[22] Kincaid JP, Fishburne RP Jr, Rogers RL, Chissom BS (1975). Derivation of New Readability Formulas (Automated Readability Index, Fog Count and Flesch Reading Ease Formula) for Navy Enlisted Personnel.

[23] Aguilera A, Garza MJ, Muñoz RF (2010). Group cognitive-behavioral therapy for depression in Spanish: Culture-sensitive manualized treatment in practice. J Clin Psychol.

[24] Aguilera A, Bruehlman-Senecal E, Liu N, Bravin J (2018). Implementing group CBT for depression among Latinos in a primary care clinic. Cogn Behav Pract.

[25] Yom-Tov E, Feraru G, Kozdoba M, Mannor S, Tennenholtz M, Hochberg I (2017). Encouraging physical activity in patients with diabetes: Intervention using a reinforcement learning system. J Med Internet Res.

[26] Altman D, Doré C (1990). Randomisation and baseline comparisons in clinical trials. Lancet.

[27] Senn SJ (1989). Covariate imbalance and random allocation in clinical trials. Stat Med.

[28] Senn S (1994). Testing for baseline balance in clinical trials. Stat Med.

[29] Qian T, Yoo H, Klasnja P, Almirall D, Murphy S (2020). Estimating time-varying causal excursion effect in mobile health with binary outcomes. Biometrika.

[30] Cohen J (1988). The effect size index: d. Statistical Power Analysis for the Behavioral Sciences. 2nd edition.

[31] Liu G, Liang K (1997). Sample size calculations for studies with correlated observations. Biometrics.

[32] Korinek EV, Phatak SS, Martin CA, Freigoun MT, Rivera DE, Adams MA, Klasnja P, Buman MP, Hekler EB (2018). Adaptive step goals and rewards: A longitudinal growth model of daily steps for a smartphone-based walking intervention. J Behav Med.

[33] Xu J, Yan X, Figueroa C, Williams J, Chakraborty B Multi-level micro-randomized trial: Detecting the proximal effect of messages on physical activity. ArXiv..

[34] Aguirre TM, Koehler AE, Joshi A, Wilhelm SL (2018). Recruitment and retention challenges and successes. Ethn Health.

[35] Medina-Ramirez P, Calixte-Civil P, Meltzer LR, Brandon KO, Martinez U, Sutton SK, Meade CD, Byrne MM, Brandon TH, Simmons VN (2020). Comparing methods of recruiting Spanish-preferring smokers in the United States: Findings from a randomized controlled trial. J Med Internet Res.

[36] Rabbi M, Klasnja P, Choudhury T, Tewari A, Murphy S, Baumeister H, Montag C (2019). Optimizing mHealth interventions with a bandit. Digital Phenotyping and Mobile Sensing. Studies in Neuroscience, Psychology and Behavioral Economics.

[37] Triantafyllidis AK, Tsanas A (2019). Applications of machine learning in real-life digital health interventions: Review of the literature. J Med Internet Res.

[38] Nahum-Shani I, Smith SN, Spring BJ, Collins LM, Witkiewitz K, Tewari A, Murphy SA (2018). Just-in-time adaptive interventions (JITAIs) in mobile health: Key components and design principles for ongoing health behavior support. Ann Behav Med.

[39] O'Connor S, Hanlon P, O'Donnell CA, Garcia S, Glanville J, Mair FS (2016). Barriers and facilitators to patient and public engagement and recruitment to digital health interventions: Protocol of a systematic review of qualitative studies. BMJ Open.

