# Supplementary material for: A Text Messaging Intervention for Coping With Social Distancing During COVID-19 (StayWell at Home): Protocol for a Randomized Controlled Trial
Source: JMIR Res Protoc. 2021 Jan 14;10(1):e23592. doi: 10.2196/23592 (PMC7813560; doi:10.2196/23592)
Supplement: Multimedia Appendix 1 [file resprot_v10i1e23592_app1.docx]

**University of California at Berkeley**

**Consent to Participate in Research**

CPHS# 2020-04-13162

**Key Information**

- You are being invited to participate in a research study. Participation in research is completely voluntary.
- The purpose of the study is to help people deal with challenging times, like the current COVID-19 pandemic, by sending helpful text messages.
- The study will take a total of one hour of your time. You will be asked to fill in an online questionnaire before the start of the study, receive text-messages for 60 days and respond to some of these messages, and fill in an online questionnaire at the end of the study.
- Risks and/or discomforts may include the risk breach of confidentiality and some of the research questions may make you uncomfortable or upset. You are free to skip/not answer any question that you do not wish to answer and are free to end participation /exit the questionnaires at any time.
- This tool may improve your mood and benefit your health. However, you may not directly benefit from the research. We may gain knowledge trough this study about the use of technology for helping people improve their mood during difficult times.

**Introduction and Purpose**

My name is Caroline Figueroa. I am a postdoctoral researcher at the University of California, Berkeley working with my faculty advisor, Professor Aguilera in the School of Social Welfare. We would like to invite you to take part in our research study, which concerns a study to help people deal with challenging times, like the current COVID-19 pandemic, by sending supportive text messages. We aim to include a maximum of 1000 people in this research study.

**Procedures**

If you agree to participate in our research, we will ask you to complete the attached online questionnaire. The questionnaire will involve questions about demographics, socioeconomic status, baseline health, physical activity, mobile technology familiarity and utilization and psychological concepts such as anxiety and depression and should take about 20 minutes to complete. We will also ask for your cell phone number so that we can send you text messages from the program.

We will enroll you in a 60-day text-messaging program to help you deal with the current effects of the pandemic. These text-messages will include tips about positive activities and coping skills to deal with worries and stress. About 50% of these messages are framed as a question, asking you to reflect on your thoughts or undertake pleasurable activities. You can respond to these messages with your reflections or plans as you wish, but this is not mandatory.

You will receive one of these messages per day between 10:00 am and 6:00 pm. You will also receive a message asking you to rate your mood on a scale of 1-9 up once a day, 3 hours after you receive a tip message. We ask you to respond to these messages, but this is not mandatory.

The approximate amount of time that you will spend responding to text messages will be about one minute per message or less. If you want to stop receiving any messages, reply STOP when you receive a text message.

At the end of 60 days, we will ask you to complete a final online questionnaire that will take about 20 minutes. A link for this online questionnaire will be sent to you through text message.

**Benefits**

This tool may improve your mood and benefit your health. However, you may not directly benefit from the research. We hope that we will gain knowledge trough this study about the use of technology for helping people improve their mood during difficult times. This can be applied to future interventions seeking to employ text messaging. Results from this study will help guide new studies on mood management.

**Risks/Discomforts**

You may choose either to take part or not to take part in the study. If you decide to take part in this study, you may leave the study at any time. You may also choose not to answer any question in the interviews that you do not wish to answer and still receive full compensation for your participation.

Some of the research questions may make you uncomfortable or upset. You are free to skip/not answer any question that you do not wish to answer and are free to end participation /exit the questionnaires at any time.

You can protect your privacy by clearing your browser’s history, cache, cookies, and other browsing data. (Warning: This will log you out of online services.)

As with all research, there is a chance that confidentiality could be compromised; however, we are taking precautions to minimize this risk.

**Confidentiality**

Your study data will be handled as confidentially as possible. If results of this study are published or presented, individual names and other personally identifiable information will not be used*.*

A description of this clinical trial will be available on [http://www.ClinicalTrials.gov](http://www.clinicaltrials.gov/). This Web site will not include information that can identify you. At most, the Web site will include a summary of the results. You can search this Web site at any time.

To minimize the risks to confidentiality, we will do the following:

All data collected from your interviews will be kept within the research team. Identifiers on your research records will be removed and will be replaced with a code where investigators will have a key that links codes to identifiers. The server receiving your responses to text messages we will send you is hosted behind a University of California San Francisco (UCSF) firewall in a secure location, subject to healthcare-grade security measures, including strict firewalls, intrusion detection, and active monitoring by study and University staff.  Only the research team will have access to your study records. We will keep your study data as confidential as possible.

When the research is completed, the maximum retention period of identified data will be a period of 5 years. The maximum retention period for the key that links identifiers to codes will be kept up to 5 years after questionnaires are collected. De-identified data will be retained indefinitely for use in possible research done by ourselves or others.

Your personal information may be released if required by law. Authorized representatives from the following organizations may review your research data for purposes such as monitoring or managing the conduct of this study:

- University of California

Identifiers might be removed from the identifiable private information. After such removal, the information could be used for future research studies or distributed to other investigators for future research studies without additional informed consent from the subject or the legally authorized representative.

**Compensation**

You will not receive compensation for your participation in the baseline questionnaire at the beginning of the study. You will receive a $20 amazon gift card for the exit questionnaire at the end of the study, which will be sent to you via text-message within two weeks of completing the surveys. Thus, the maximum compensation you will receive is $20, in amazon gift cards for participating in the exit questionnaire after the 60-day study. You will not be compensated for receiving and responding to the text-messages.

**Rights**

**Participation in research is completely voluntary**. You are free to decline to take part in the project. You can decline to answer any questions and are free to stop taking part in the project at any time. Whether or not you choose to participate, to answer any particular question, or continue participating in the project, there will be no penalty to you or loss of benefits to which you are otherwise entitled.

**Questions**

If you have any questions about this research, please feel free to contact me/us. You can reach me, Caroline Figueroa at c.a.figueroa@berkeley.edu. Or you may contact Adrian Aguilera, Principal Investigator, at 415-206-6166.

If you have any questions about your rights or treatment as a research participant in this study, please contact the University of California at Berkeley’s Committee for Protection of Human Subjects at 510-642-7461, or e-mail [subjects@berkeley.edu](mailto:subjects@berkeley.edu).

If you agree to participate in this research study, and if you are over 18 years old, please print a copy of this page (or save as PDF and send to yourself via email) to keep for future reference and click on the “Accept” button below.

O I accept and I am over 18 years old

O I do not accept

O I am under 18 years old
